# Genome‐wide screening of abberant methylated drivers combined with relative risk loci in bladder cancer

**DOI:** 10.1002/cam4.2665

**Published:** 2019-12-03

**Authors:** Chuanjie Zhang, Kangjie Shen, Yuxiao Zheng, Feng Qi, Jun Luo

**Affiliations:** ^1^ Department of Urology Ruijin Hospital School of Medicine Shanghai Jiaotong University Shanghai China; ^2^ First Clinical Medical College of Nanjing Medical University Nanjing China; ^3^ Department of Urology Jiangsu Cancer Hospital Jiangsu Institute of Cancer Research Affiliated Cancer Hospital of Nanjing Medical University Nanjing China; ^4^ Department of Urology Shanghai Fourth People's Hospital affiliated to Tongji University School of Medicine Shanghai China

**Keywords:** bladder cancer (BCa), genome‐wide, methylation‐driven genes (MDGs), prognosis

## Abstract

**Background:**

To explore important methylation‐driven genes (MDGs) and risk loci to construct risk model for prognosis of bladder cancer (BCa).

**Methods:**

We utilized TCGA‐Assembler package to download 450K methylation data and corresponding transcriptome profiles. MethylMix package was used for identifying methylation‐driven genes and functional analysis was mainly performed based on ConsensusPathDB database. Then, Cox regression method was utilized to find prognostic MDGs, and we selected 17 hub genes via stepwise regression and multivariate Cox models. Kruskal‐Wallis test was implemented for comparisons between risk with other clinical variables. Moreover, we constructed the risk model and validated it in http://www.ncbi.nlm.nih.gov/geo/query/acc.cgi?acc=GSE13507. Gene set enrichment analysis was performed using the levels of risk score as the phenotype. Additionally, we further screened out the relative methylation sites associated with the 17 hub genes. Cox regression and Survival analysis were conducted to find the specifically prognostic sites.

**Results:**

Two hundred and twenty‐eight MDGs were chosen by ConsensusPathDB database. Results revealed that most conspicuous pathways were transcriptional mis‐regulation pathways in cancer and EMT. After Cox regression analysis, 17 hub epigenetic MDGs were identified. We calculated the risk score and found satisfactory predictive efficiency by ROC curve (AUC = 0.762). In the validation group from http://www.ncbi.nlm.nih.gov/geo/query/acc.cgi?acc=GSE13507, 17 hub genes remained higher predictive value with AUC = 0.723 and patients in high‐risk group. Meanwhile, Kruskal‐Wallis test revealed that higher risk score correlated with a higher level of TNM stage, tumor grade, and advanced pathological stages. Then, identified 38 risk methylated loci that highly associated with prognosis. Last, gene set enrichment analysis revealed that high‐risk level of MDGs may correlate with several important pathways, including MAPK signaling pathway and so on.

**Conclusion:**

Our study indicated several hub‐MDGs, calculated novel risk score and explored the prognostic value in BCa, which provided a promising approach to BCA prognosis assessment.

## Graphical abstract

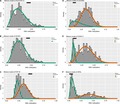

## 1 INTRODUCTION

Bladder cancer (BCa) is the 2nd most common form of malignancy in urological system worldwide.^1^ In 2019, the estimated respective new cases and new death are 80 470 and 17 670 in the United States reported by the American Cancer Society.^2^ With incidence and mortality rates of 9.6 and 3.2 per 100 000, respectively, BCa is approximately four times common in males than in females.^1^ According to the extent of tumor invasion, about three‐quarters of BCa patients classified as nonmuscle‐invasive BCa (NMIBC) and the remainder had muscle‐invasive BCa (MIBC) when initial diagnosed.^3^ Despite the comprehensive treatments of surgical treatment, radiotherapy, chemotherapy, and immunotherapy, BCa is still a serious challenge due to high recurrence and mortality.^4^, ^5^

Apart from certain occupational chemical explosion, water pollution, and tobacco smoking, genetic material mutation is one of the main risk factors for BCa.^6^, ^7^, ^8^ However, the molecular regulation mechanism of BCa remains confused. Both DNA and histone methylation are an essential epigenetic regulation mechanism that has been studied intensively in recent decades.^9^, ^10^, ^11^, ^12^, ^13^ Tumor suppressor genes might be hypermethylated and ultimately silenced by abnormal methylation genes in CpG islands located in or near the genomic promoter region. It is noteworthy that DNA methylation shows the enormous difference between NMIBC and MIBC. For instance, in NMIBC hypomethylation in non­CpG islands is more common, while widespread promoter hypermethylation is identified more in MIBC.^14^, ^15^ This proves in some way that DNA methylation changes might lead to different clinical and pathological characteristics in BCa. However, the DNA methylation regulation effect and mechanism in BCa have not been fully elucidated.

Recently, the accelerated development of bioinformatics has been applied in malignant tumor research benefit from the improvement of high‐throughput sequencing technology, emergence of new statistical algorithms and the consummation function of public databases. The Cancer Genome Atlas (TCGA) database and Gene Expression Omnibus (GEO) database provide great convenience for systematic collection of clinical, pathological, and biological data from patients with malignancies.^16^, ^17^ In addition, Olivier Gevaert et al invented Methylmix, which is an original computational algorithm applied in correlation analysis between abnormality of DNA methylation and predict transcription.^18^ This provides a new technical means for researching DNA methylation in malignant tumors.

In this study, we filtrated methylation‐driven genes (MDGs) extracted from gene methylation profiling datasets and gene expression profiling datasets. Then the analyzed data of BCa patients from TCGA and GEO database were integrated in order to identify hub MDGs using biological algorithm. Finally, we combined selected MDGs with relative risk loci for predicting prognosis in BCa.

## 2 METHODS AND MATERIALS

### 2.1 Data acquisitions and integrative analysis

We obtained RNA‐seq data of transcriptome profiles of BCA from TCGA database (https://portal.gdc.cancer.gov/), including 414 tumor tissues and 10 normal tissues. We used edgeR package^19^ to normalize the raw data. Moreover, we utilized the tool of TCGA‐Assembler to download the DNA methylation data (419 BCA and corresponding 21 normal samples) based on the Illumina Infinium Human Methylation 450 platform, which detecting over 480 K human genome methylated sites. Limma package was applied to do the normalization.^20^ Level three methylation data were in form of β value, representing the ratio of the methylation probe data vs total probe intensities. Then, the average DNA methylation value for all CpG sites correlated with a gene was calculated and merged into a matrix with the function of TCGA‐Assembler. What is more, we subsequently extracted corresponding clinical features from 412 BCA patients via TCGA portal including age, gender, pathological stage, tumor grade, TNM stage, and survival data. Specific baseline characteristics of BCA patients are shown in Table 1 and Table S1.

**Table 1:** Clinical characteristics of all eligible 570 BLCA patients from TCGA cohort and http://www.ncbi.nlm.nih.gov/geo/query/acc.cgi?acc=GSE13507

Variables	Training group	validation group	Entire group
	(n = 405)	(n = 165)	(n = 570)
Status			
Alive	249 (61.5)	96 (58.2)	345 (56.7)
Dead	156 (38.5)	69 (41.8)	225 (43.3)
Age	68 ± 10.57	65.18 ± 11.97	67.19 ± 11.06
Gender			
Female	106 (26.2)	30 (18.2)	136 (23.9)
Male	299 (73.8)	135 (81.8)	434 (76.1)
Race			
White	334 (82.5)	NA	NA
Asian	43 (10.6)	NA	NA
Black or African	28 (6.9)	NA	NA
AJCC‐T			
T0/Ta	1 (0.2)	24 (14.5)	25 (4.4)
T1	3 (0.8)	80 (48.5)	83 (14.6)
T2	117 (28.9)	31 (18.8)	148 (25.9)
T3	193 (47.7)	9 (5.5)	212 (37.2)
T4	58 (14.3)	11 (6.7)	69 (12.1)
Unknown	33 (8.1)	0 (0.0)	33 (5.8)
AJCC‐N			
N0	235 (58.0)	149 (90.3)	384 (67.4)
N1	46 (11.4)	8 (4.9)	54 (9.5)
N2	75 (18.5)	6 (3.6)	81 (14.2)
N3	7 (1.7)	1 (0.6)	8 (1.4)
Unknown	42 (10.4)	1 (0.6)	43 (7.5)
AJCC‐M			
M0	195 (48.1)	158 (95.8)	353 (61.9)
M1	11 (2.7)	7 (4.2)	18 (3.2)
Mx	199 (49.2)	NA	NA
Pathologic_stage			
I & II	130 (32.1)	NA	NA
III & IV	273 (67.4)	NA	NA
Unknown	2 (0.5)	NA	NA
Tumor_grade			
G1/G2	20 (5.0)	105 (63.6)	125 (21.9)
G3/G4	382 ( 94.3)	60 (36.4)	442 (77.6)
Unknown	3 (0.7)	NA	NA
MDGs risk score			
Low	203 (50.1)	82 (50.0)	285 (50.0)
High	202 (49.9)	83 (50.0)	285 (50.0)

**Figure 1:**
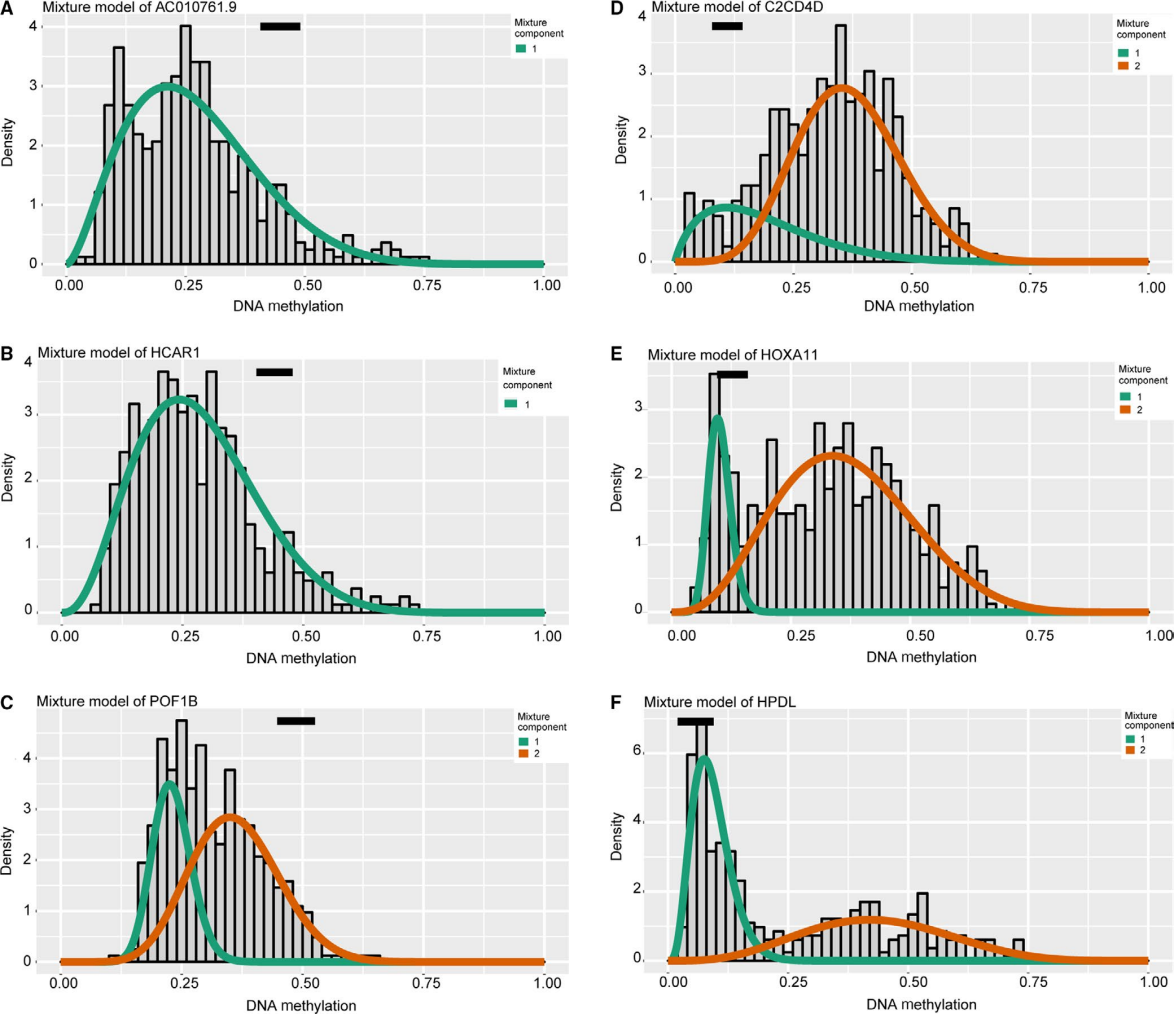
Illustration of the mixture model to screen MDGs via MethylMix package. Distribution of methylated status in tumor samples was shown by histogram, in which two lines represented the two components. The black line revealed the methylation data in normal tissues. A‐C, Top three hypermethylated signatures. D‐F, Identification of top three hypomethylated genes

MethylMix package was designed to deal with analysis of methylation data with RNA‐seq profiles, especially detecting methylation alterations that were associated with gene expression.^18^ We experienced three steps during the algorithm as following: firstly, methylation events that lead to alterations of gene expression were selected that only the genes passed the correlation cutoff value of correlation coefficients = −0.3 and adjust *P* value = .05. Second, the methylated state of one gene was defined across multiple samples using a β mixture model. Last, the comparison of methylation levels in BCA and corresponding normal samples was conducted using the Wilcoxon rank sum test (Figure 1).

### 2.2 Cluster analyses and enriched pathways of methylated drivers

Differentially expressed divers with methylated alterations were defined by MethylMix package and performed the cluster analysis between tumor vs normal tissues using pheatmap package. In addition, we applied the ConsensusPathDB database to further assess the potential enriched pathways using the imputed driven‐genes list. ConsensusPathDB was publicly comprehensive biological resources mainly integrating interaction networks and we selected Humancyc, Reactome, Kegg, Smpdb, Wikipathways, Signalink, and Biocarta for subsequent analysis with *P* value cutoff of .05.^21^

### 2.3 Construction and evaluation of epigenetic risk score based on MDGs

We conducted the univariate Cox regression analysis to select prognostic MDGs with *P* < .05 as the threshold. Stepwise regression analysis was performed to select 17 hub MDGs with the minimum Akaike information criterion (AIC) value. Meanwhile, we utilized the multivariate Cox method to obtain the *β* value of hub genes, which was the coefficient of each variable and represented the respective weight. Hazard ratio and 95% confidence interval (95% CI) were calculated and shown in forest plot by survminer package. Then, risk score based on the epigenetic MDGs could be calculated as the follows: Risk score = Ʃ (*β*_i_ × Exp_i_) (i = 17), where Exp represented the expression data of 17 hub genes. Based on this, 405 BCA patients from TCGA cohort can be divided into high‐ and low‐ risk groups using the median risk score as the cutoff. Importantly, we further used the receiver operating characteristic curve (ROC) to evaluate the predictive efficiency of MDGs with survivalROC package.^22^ Significant difference between high‐ and low‐ risk groups was assessed by Kaplan‐Merier analysis using survival package.

### 2.4 Association analyses between MDGs and clinical characteristics

Since BCA patients in high‐risk group revealed worse survival prognosis than that in low‐risk group, we invented to further investigated the potential associations between MDGs signature with traditional clinical characteristics. We downloaded and collated clinical variables from 405 patients in TCGA cohort with complete information consisted of AJCC‐TNM stage, tumor grade, as well as pathological stage, which was all independent clinical risk factors. We merged the risk score with other clinical variables with the same id numbers for subsequent analysis (Table S4). Kruskal‐Wallis (K‐W) test was utilized to assess the significant difference between level of risk score across multiple groups in respective clinical features with statistical cutoff of *P* < .05.

### 2.5 Validation of MDGs signature in an independent population

To evaluate the predictive power of 17 MDGs signatures, we obtained 165 BCA patients with corresponding expression data and clinical information in http://www.ncbi.nlm.nih.gov/geo/query/acc.cgi?acc=GSE13507 from GEO database (https://www.ncbi.nlm.nih.gov/geo/). We selected the same 17 hub genes and constructed the risk formula based on multivariate Cox model using survival package. Importantly, the cutoff was set as the same as the TCGA‐BLCA cohort and we classified the patients into low and high subgroups. The AUC of the ROC curve was calculated with survivalROC package for assessing the power of 17 MDGs signature in OS prediction and survival curve was drawn by a survival package.

### 2.6 Analysis of genes and methylated loci associated with OS

To further detect specific methylated sites in 17 hub MGDs, we extracted the all methylation data correlated with 17 MDGs in 440 files from TCGA using the Perl scripts. We merged the value of 207 methylated sites into one matrix and conducted univariate Cox analysis to select potential risk methylated loci (Table S3). Survival analysis was then performed to assess the difference between hypo‐ and hypermethylation state of specific sites (Figure S3). Additionally, a joint survival analysis was conducted to further evaluate key MDGs signature associated with prognosis in BCA patients, where we combined the methylation levels and expression data of one gene. The conjoint analysis was also performed by the survival package.

### 2.7 Gene set enrichment analysis (GSEA)

GSEA software was downloaded from the GSEA home (http://software.broadinstitute.org/gsea/index.jsp) and work based on JAVA 8 platforms (https://www.oracle.com/java/).^23^ We classified the 405 BCA patients into 202 high‐ and 203 low‐risk group, which was used as the phenotypes. Then, we selected the “c2.cp.kegg.v6.2.symbols.gmt gene sets” are obtained from the MSigDB (http://software.broadinstitute.org/gsea/downloads.jsp) as the reference gene sets. Furthermore, we defined the false discovery rate (FDR) < 0.25, |enriched score| >0.35, and gene size ≥35 as the cutoff criteria.

### 2.8 Statistical analyses

The Kruskal‐Wallis (K‐W) test was a nonparametric test and mainly conducted for three or more dataset. Besides, the Wilcoxon rank sum test was utilized to compare the methylation state in tumor vs normal tissues. Pearson correlation coefficients were calculated. The survival analysis was mainly based on the survival package. All statistical analysis was performed by R studio (version 3.5.3). A *P* value <.05 was considered to be of statistical significance.

## 3 RESULTS

### 3.1 Identification of methylated driven genes

**Figure 2:**
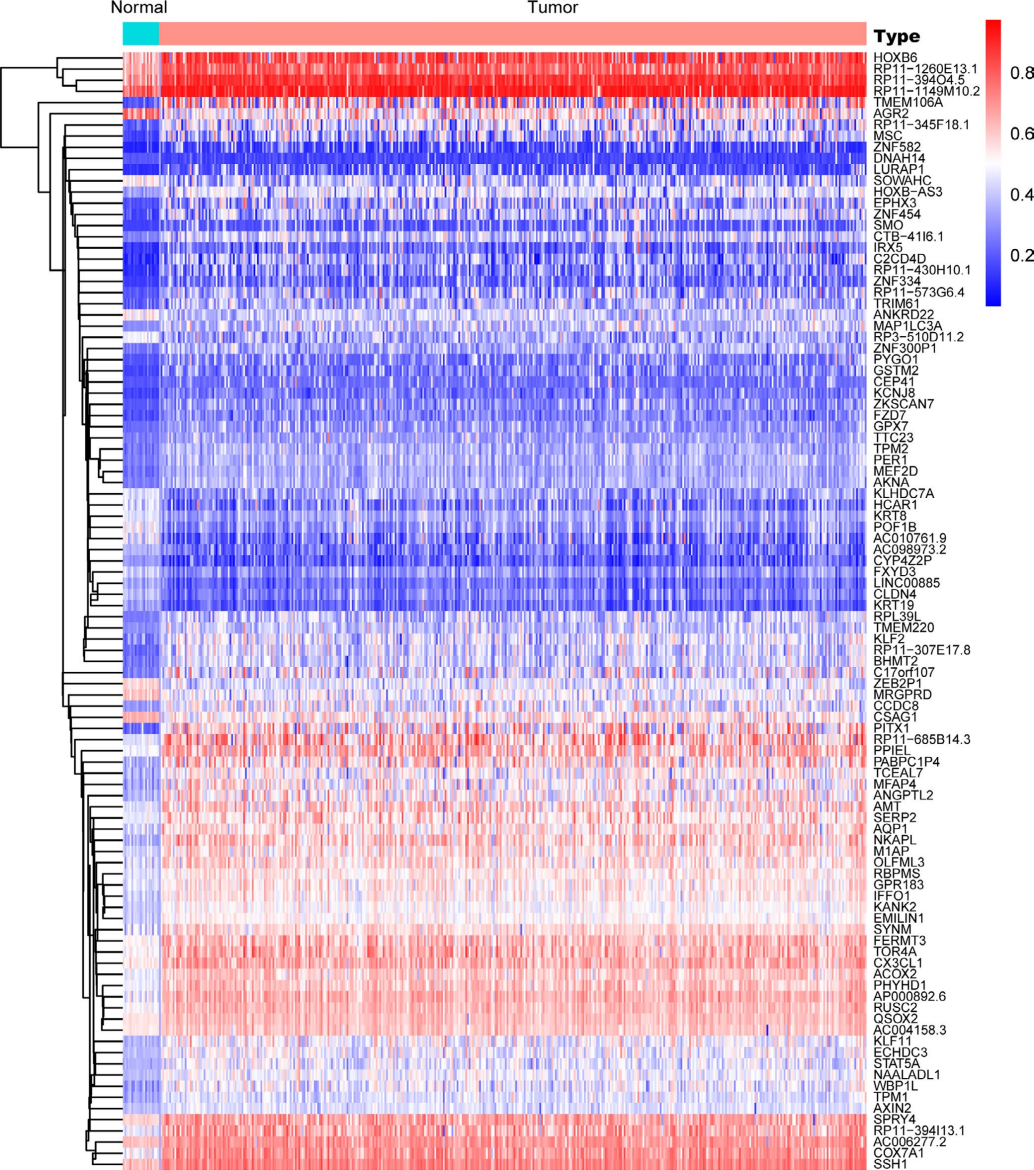
Heatmap of top 100 MDGs was drawn to reveal differential distribution of methylated state, where the colors of blue to red represented alterations from hypomethylation to hypermethhylation

Raw transcriptome data of 414 BCA with 10 normal samples were normalized via edgeR package, and we identified 8898 differentially expressed genes with cutoff of FDR <0.05. Meanwhile, we prepared and normalized the methylation profiles of 419 BCA with corresponding 21 normal samples through limma package. Then, the results from the MethylMix package revealed the correlations between methylation state with genes expression, mixture models, as well as the methylation difference between BCA and normal tissues across multiple samples. A total of 228 genes were defined as the epigenetic drivers with |logFC| > 0, |Cor| > 0.3, and *P* < .05 (Table S2). We showed the top hypo‐ and hypermethylation of drivers in Figure 1. We selected top 100 genes to show the difference of methylated level between tumor and normal tissues, which were illustrated in heatmap with pheatmap package in Figure 2.^20^

### 3.2 Functional enriched pathways

**Figure 3:**
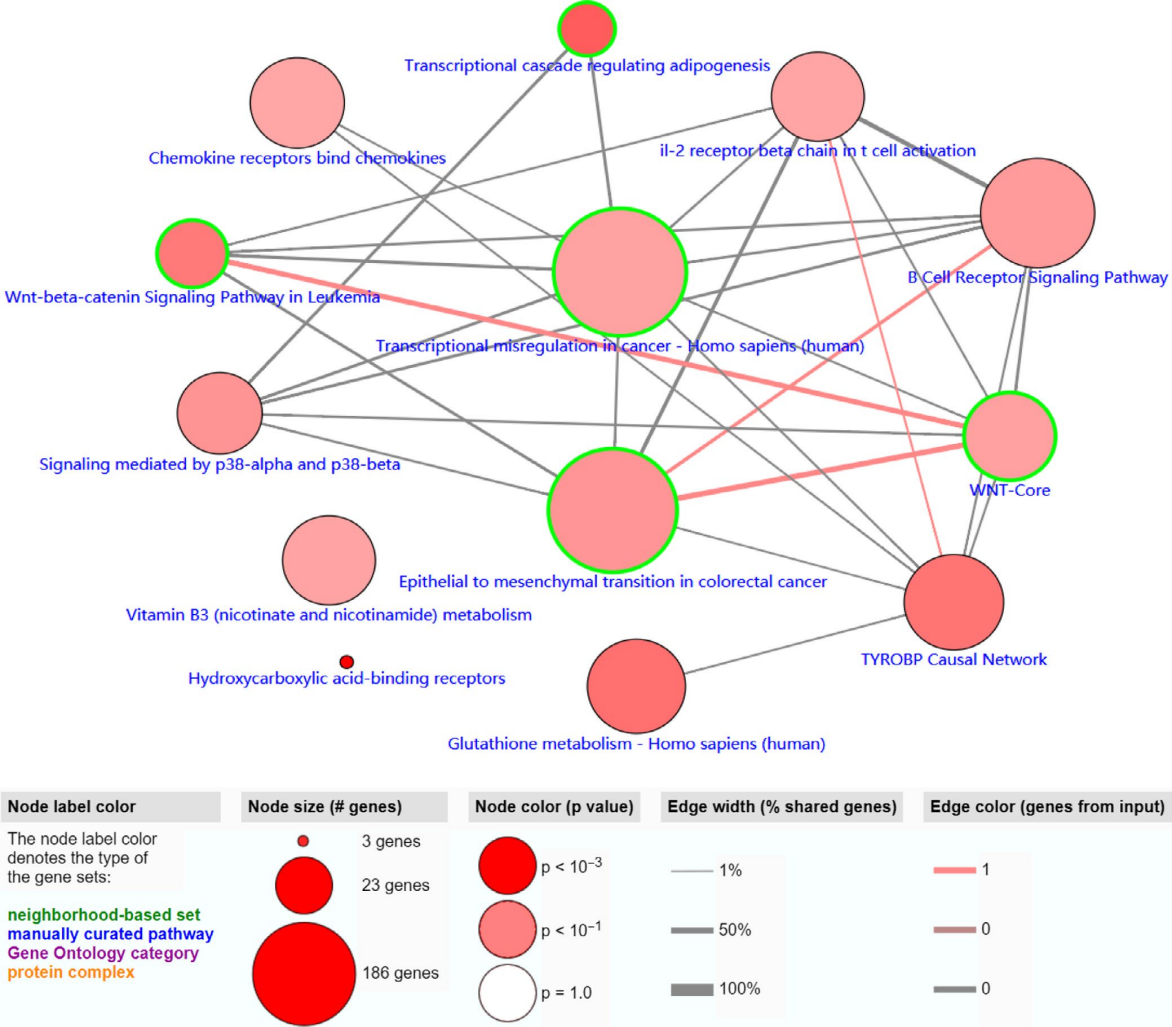
Functional pathway analysis for 228 MDGs based on ConsensusPathDB database. Transcriptional misregulation in cancer, the MAPK signaling pathway, the Wnt signaling pathway, cell cycle, as well as other cancer‐related pathways enriched significantly in our analysis

Since we obtained 228 MDGs found to be statistically significant via MethylMix package, we intended to further explore the potential pathways that the divers might be involved in. We conducted the functional enriched analysis across multiple database in ConsensusPathDB and selected several significant biological pathways. The most conspicuous pathways were transcriptional misregulation pathways in cancer and epithelial to mesenchymal transition (EMT). In addition, there were other notable pathways including Wnt signaling crosstalk, glutathione metabolism, transcriptional cascade regulating pathway, as well as B‐cell receptor signaling pathways. We selected several vital pathways to exhibit in Figure 3, and the other top 40 pathways were shown in Table 2.

**Table 2:** Functional analysis of MDGs based on ConsensusPathDB database

Enriched pathway	*P* value	source
Signaling mediated by p38‐alpha and p38‐beta	.024851855	PID
Wnt‐beta‐catenin Signaling Pathway in Leukemia	.01023165	Wikipathways
WNT‐Core	.037948147	Signalink
Glutathione metabolism	.00704807	KEGG
Signaling events mediated by HDAC Class III	.030398536	PID
B Cell Receptor Signaling Pathway	.031370148	Wikipathways
Fas Ligand pathway and Stress induction of Heat Shock Proteins regulation	.036385809	Wikipathways
Transcriptional cascade regulating adipogenesis	.003598992	Wikipathways
Hydroxycarboxylic acid‐binding receptors	.000144888	Reactome
Transcriptional misregulation in cancer	.040866181	KEGG
Chemokine receptors bind chemokines	.047839785	Reactome
FTO Obesity Variant Mechanism	.001321732	Wikipathways
White fat cell differentiation	.001423078	Wikipathways
Epithelial to mesenchymal transition in colorectal cancer	.025906124	Wikipathways
TYROBP Causal Network	.008528042	Wikipathways
Bopindolol Action Pathway	.008924337	SMPDB
Timolol Action Pathway	.008924337	SMPDB
Carteolol Action Pathway	.008924337	SMPDB
Bevantolol Action Pathway	.008924337	SMPDB
Practolol Action Pathway	.008924337	SMPDB
Dobutamine Action Pathway	.008924337	SMPDB
Isoprenaline Action Pathway	.008924337	SMPDB
Arbutamine Action Pathway	.008924337	SMPDB
Levobunolol Action Pathway	.008924337	SMPDB
Metipranolol Action Pathway	.008924337	SMPDB
Sotalol Action Pathway	.008924337	SMPDB
Epinephrine Action Pathway	.008924337	SMPDB
Betaxolol Action Pathway	.008924337	SMPDB
Atenolol Action Pathway	.008924337	SMPDB
Alprenolol Action Pathway	.008924337	SMPDB
Acebutolol Action Pathway	.008924337	SMPDB
Propranolol Action Pathway	.008924337	SMPDB
Pindolol Action Pathway	.008924337	SMPDB
Penbutolol Action Pathway	.008924337	SMPDB
Oxprenolol Action Pathway	.008924337	SMPDB
Metoprolol Action Pathway	.008924337	SMPDB
Esmolol Action Pathway	.008924337	SMPDB
Bisoprolol Action Pathway	.008924337	SMPDB
Bupranolol Action Pathway	.008924337	SMPDB
Nebivolol Action Pathway	.008924337	SMPDB

### 3.3 Construction and validation of risk scores based on MDGs signatures

**Figure 4:**
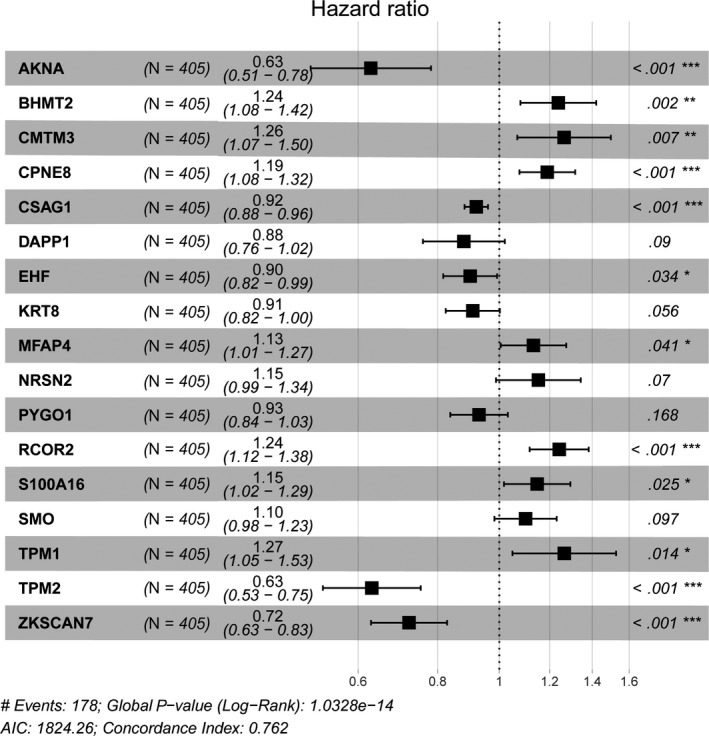
Forest plot of 17 hub MDGs in TCGA cohort. The Concordance Index and the Minimum of AIC were shown at the left bottom of the picture

**Figure 5:**
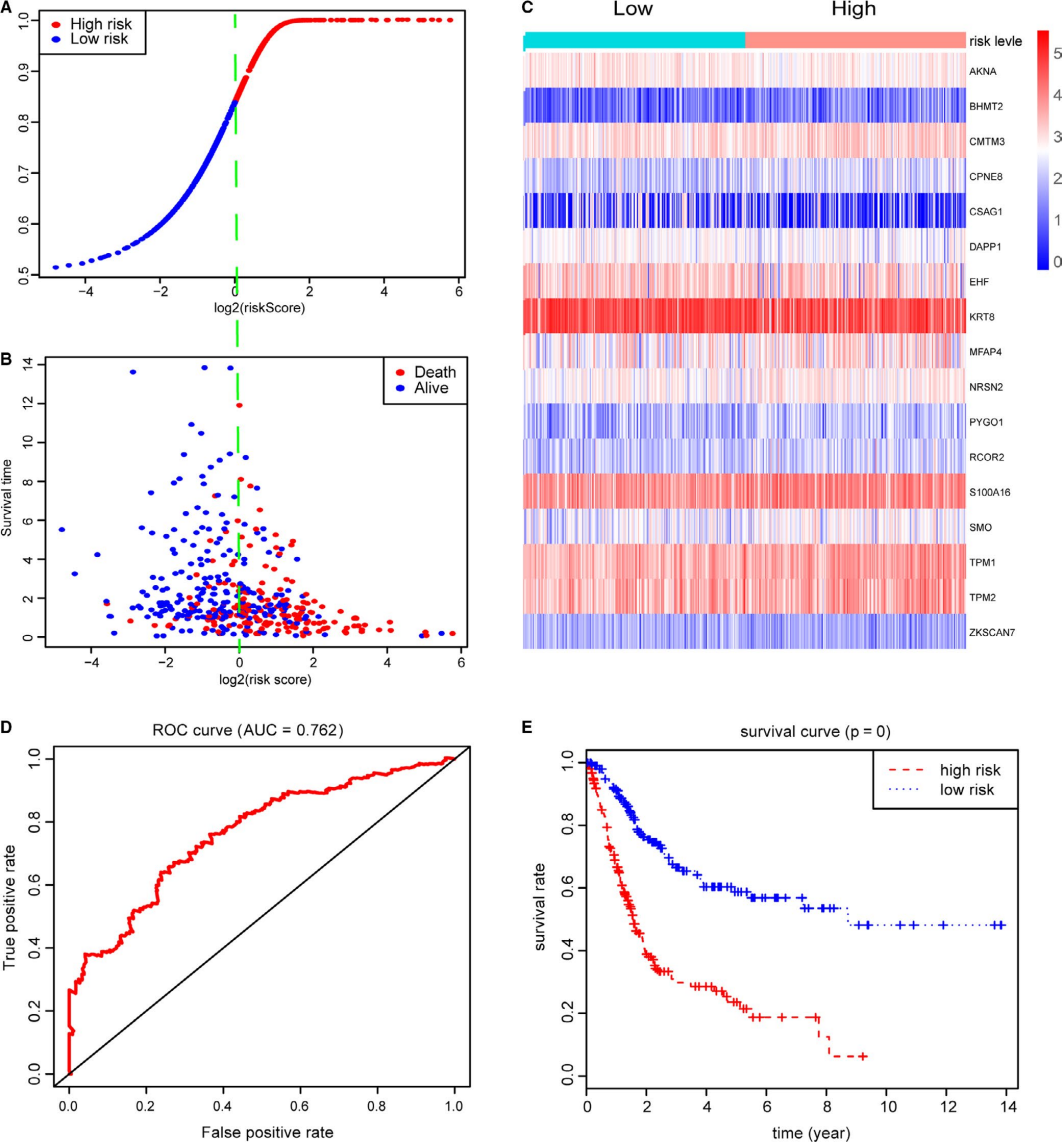
Construction of risk scores based on 17 hub MDGs. A‐B, Distribution of vital status of 405 BCA patients in high‐ and low‐risk groups. C, Heatmap of expression levels for hub MDGs in patients with high‐ and low‐risk score levels. D, ROC curve for 3‐year prediction was utilized for assessing the prognostic values of risk score with AUC = 0.762. E, Bca patients in high‐risk group revealed worse survival outcomes than that in low‐risk groups

We utilized the univariate Cox regression analysis to identify 64 genes associated with survival outcomes with *P* < .05 (Table S5). Then, the stepwise regression model was performed to select a robust signature that was the most frequent combinations, where 17 epigenetic divers were screened out. We showed the hazard ratio with 95% CI for each hub MDGs based on the multivariate Cox regression results in a forest plot (Figure 4). Therefore, a methylation‐based signature was established with the 17 hub MDGs. Meanwhile, the association analysis revealed that the methylation state all correlated negatively with the expression level of genes from the Pearson correlation coefficients in Figure S1. To further assess the predictive value of methylation signature in OS prediction, we calculated the risk score as following: risk score = (−0.46463)*AKNA + 0.21378*BHMT2 + 0.23461*CMTM3 + 0.17359*CPNE8 + (−0.08307)*CSAG1 + (−0.12821)*DAPP1 + (−0.10515)*EHF + (−0.09579)*KRT8 + 0.12370*MFAP4 + 0.14159*NRSN2 + (−0.07314)*PYGO1 + 0.21693*RCOR2 + 0.13725*S100A16 + 0.09522*SMO + 0.23525*TPM1 + (−0.46111)*TPM2 + (−0.32630)*ZKSCAN7. Then, we obtained the respective risk score and divided the 405 BCA into high‐risk group (n = 202) and low‐risk group (n = 203). The distribution of methylation‐based risk score and vital status of BCA patients in two groups was shown in Figure 5A,B. Moreover, the expression level of 17 genes in two groups was exhibited in heatmap (Figure 5C). The AUC of the ROC curve was 0.762 which indicating the satisfactory predictive efficiency of the risk signature (Figure 5D). The patients in high‐risk group revealed the wore prognosis in OS compared with that in low‐risk group from the Kaplan‐Merier plot with *P* < .0001 in Figure 5E.

**Figure 6:**
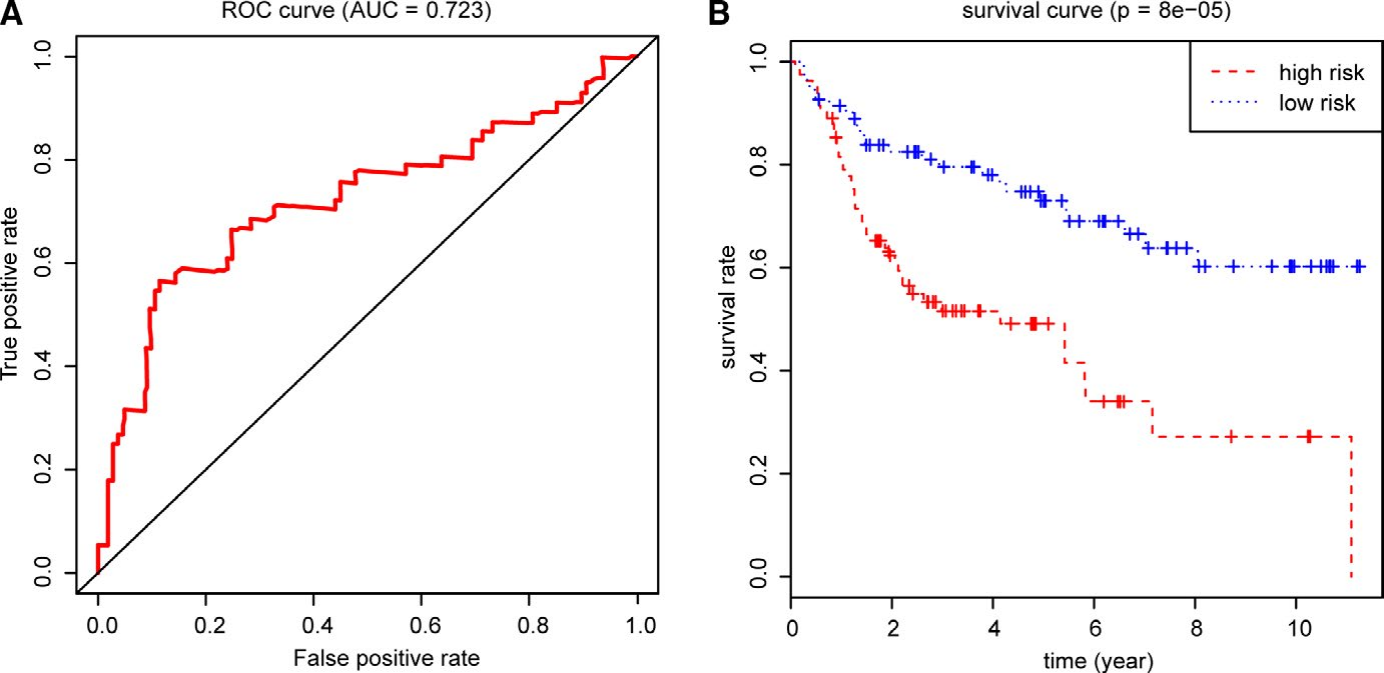
Validation of 17 hub MDGs in http://www.ncbi.nlm.nih.gov/geo/query/acc.cgi?acc=GSE13507. A, The 17‐MDGs based signature remained predictive accuracy of the ROC plot (AUC = 0.723). B, Patients in high‐risk group in http://www.ncbi.nlm.nih.gov/geo/query/acc.cgi?acc=GSE13507 stilled showed poor survival probabilities

Besides, we obtained the expression profiles with corresponding clinical information of 165 BCA patients from http://www.ncbi.nlm.nih.gov/geo/query/acc.cgi?acc=GSE13507 as an independent external validation. Using the same genes identified from the TCGA cohort, the 17‐MDGs based signature successfully stratified the 165 BCA patients into low‐ (n = 82) and high‐risk group (n = 83). Furthermore, the ROC curve was conducted and the AUC was 0.723. The log‐rank test showed the significant difference of OS in high‐ and low‐risk groups with *P* = 8e‐05, in accordance with the results from TCGA cohort (Figure 6A,B). We calculated and merged the risk score with other clinical variables in Table S4.

### 3.4 Correlation of methylation‐based signature with clinical characteristics

With the result of risk score found to be statistically significant with the OS for BCA patients, we further invented to explore the potential relationships of risk score and other independent clinical risk features. The Kruskal‐Wallis test showed that higher risk score correlated with a higher level of TNM stage, high tumor grade, as well as advanced pathological stage (Stage III and IV). The specific statistical difference in distribution of risk score across multiple groups of clinical variables was shown in Figure S2A‐E.

### 3.5 Screening of risk methylation loci with survival analysis, joint survival analysis

**Figure 7:**
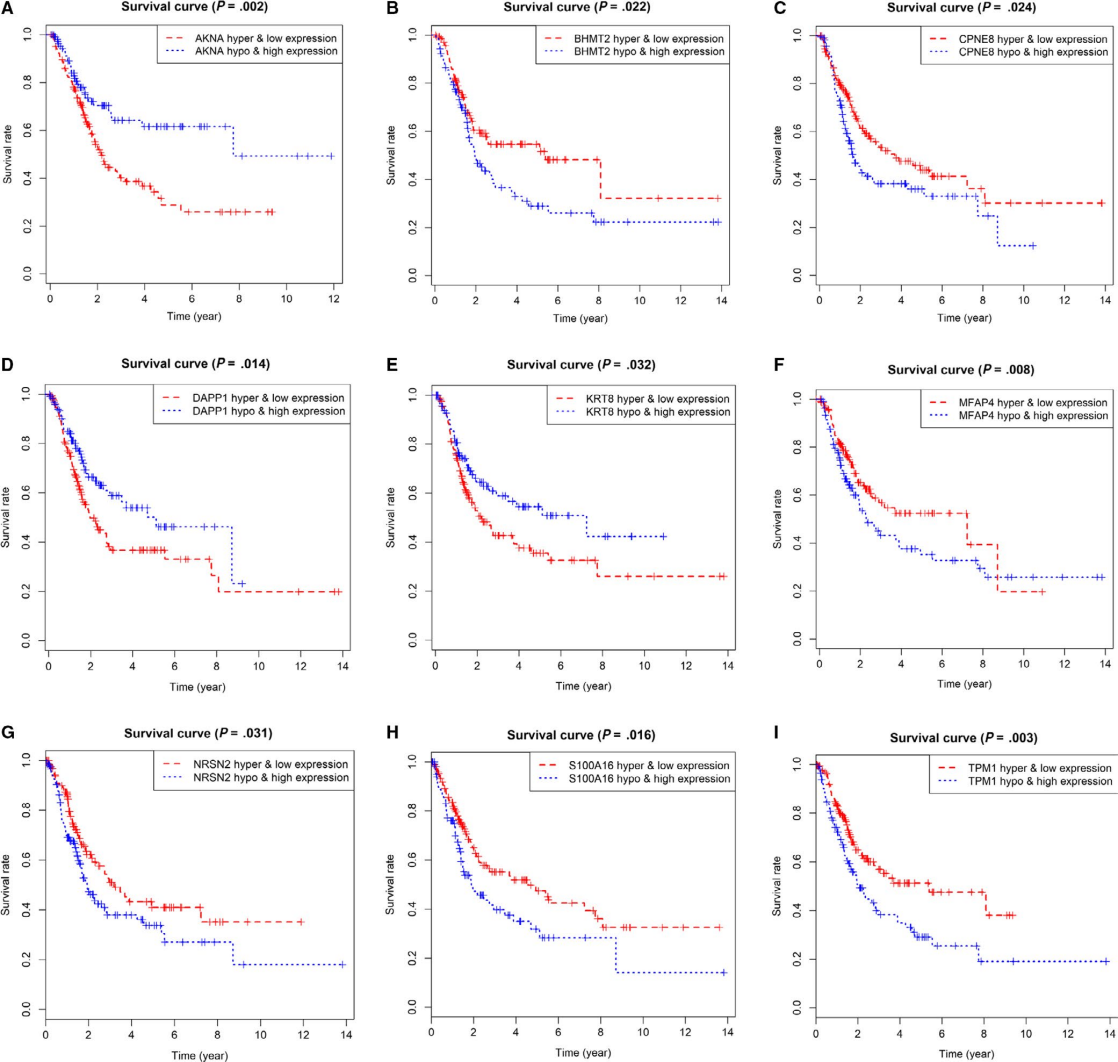
Joint survival analysis combined with methylation state and expression profiles for top 9 genes

Since we identified 17 hub MDGs associated highly with survival prognosis of BCA, we wanted to detect the significant methylation loci harboring these risk methylation genes. We extracted the β value of methylation data according to the 17 gene names from the 440 files in Table S3. A list of 207 methylation sites were screened out and the univariate Cox analysis revealed that 38 key methylation loci were associated with survival outcomes in Table 3 (*P* < .05). Meanwhile, we conducted the Kaplan‐Merier analysis and chose the plots of top 12 methylated sites in Figure S3. Additionally, a joint survival analysis found that combination of the methylation state and expression level of the 17 hub MDGs signature also correlated tightly with survival outcomes in 405 BCA patients (Figure 7).

**Table 3:** Screening of 38 prognostic risk loci associated with hub MDGs in Bca

Risk methtylated locus	Related genes	Hazard ratio	z	*P* value
cg18414381	EHF	3.9737182	3.347493733	.000815458
cg05503887	EHF	3.296494314	3.334039828	.000855944
cg00543460	TPM1	0.313566503	−3.040593266	.002361126
cg11065015	NRSN2	0.171047292	−3.031903254	.002430171
cg13836318	TPM1	0.000319606	−3.014958919	.002570141
cg24504361	KRT8	6.742218982	2.907971041	.00363782
cg14506696	DAPP1	3.366923308	2.895197969	.003789195
cg21614638	DAPP1	2.982090283	2.81620796	.00485942
cg24097814	KRT8	3.116236706	2.791702503	.005243154
cg08198488	TPM1	0.293122612	−2.782702312	.005390824
cg10397389	DAPP1	2.804089164	2.770618241	.005594998
cg01902605	BHMT2	0.156581583	−2.672356579	.007532056
cg12494355	TPM2	0.095012775	−2.660337122	.007806247
cg10843343	KRT8	5.522987956	2.648437613	.008086477
cg22235258	EHF	2.970907821	2.633435641	.008452585
cg00520135	TPM1	0.199663869	−2.632332676	.008480078
cg19460095	EHF	2.88706523	2.62182459	.008746043
cg17147440	TPM1	0.38112838	−2.608256727	.009100467
cg26357344	KRT8	2.626134721	2.58341772	.00978268
cg13107144	TPM1	0.080325594	−2.567638984	.010239373
cg06398236	DAPP1	3.783262029	2.507009355	.012175749
cg06997997	MFAP4	0.058274726	−2.502867154	.012319178
cg03790745	RCOR2	0.002665687	−2.480754698	.013110456
cg07814567	DAPP1	2.30196051	2.478978727	.013175916
cg10403394	TPM1	0.231823345	−2.390524206	.01682434
cg21083175	RCOR2	0.188494833	−2.368506257	.017860078
cg03400060	BHMT2	0.228984691	−2.360536791	.018248508
cg14260530	AKNA	0.162252883	−2.333684214	.019612263
cg18560551	EHF	2.009472208	2.329803583	.019816535
cg21867345	ZKSCAN7	2.078224581	2.182145936	.029098764
cg13969788	TPM2	0.400926556	−2.158779228	.030867299
cg14022090	EHF	2.242798579	2.148000039	.031713752
cg08162426	CPNE8	2.345731899	2.132073638	.033000793
cg22619810	RCOR2	0.015622716	−2.130825555	.033103516
cg20324165	KRT8	2.762704434	2.109031651	.03494185
cg13999433	AKNA	0.137520251	−2.017220181	.043672541
cg02619205	AKNA	0.36559657	−1.976424603	.048106707
cg01835489	KRT8	2.264564818	1.964142113	.049513614

### 3.6 Enrichment of cancer‐related pathways associated with MDGs signature

**Figure 8:**
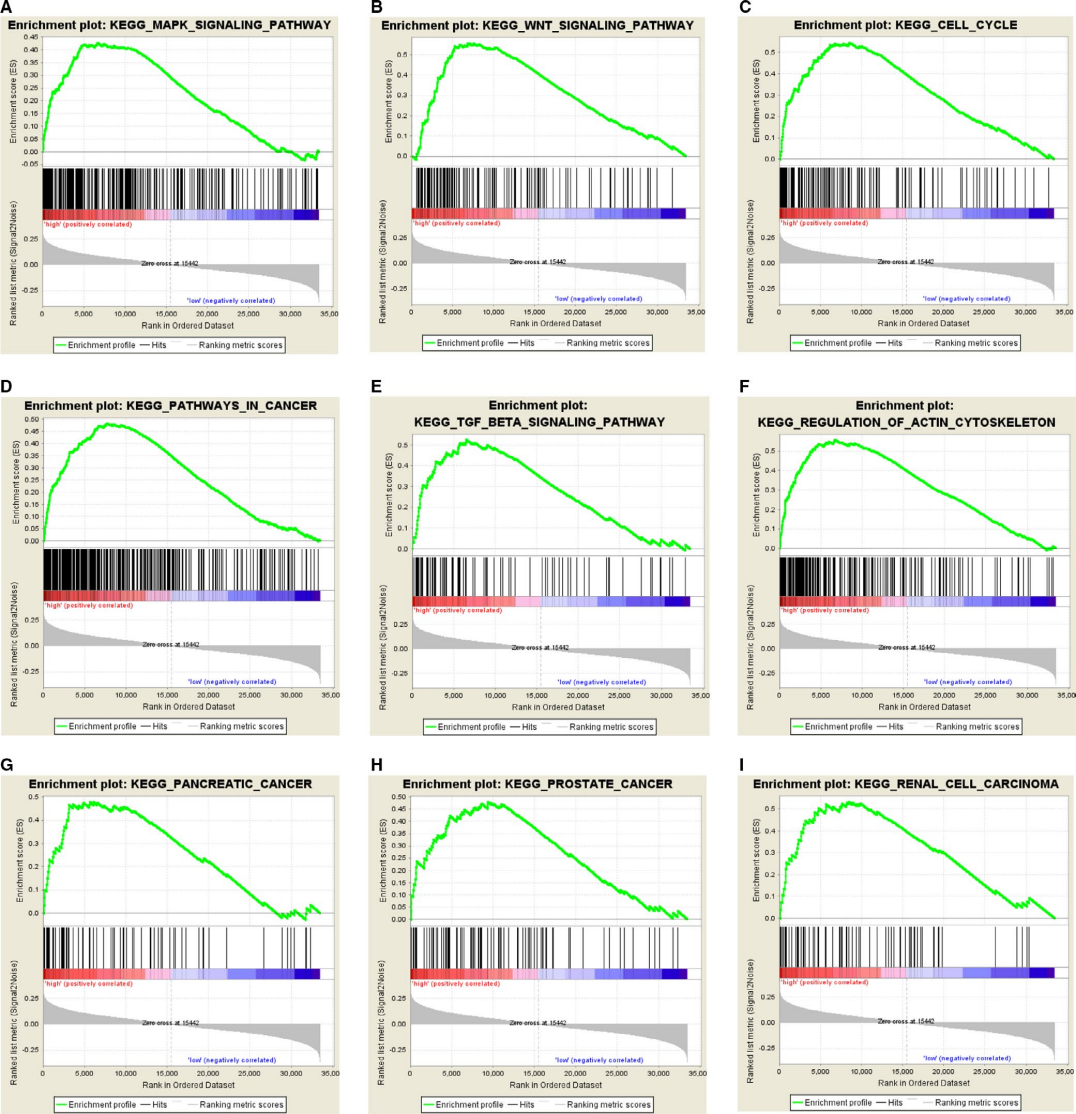
Gene set enrichment analysis for identification of the underlying pathways using risk score as the phenotype. A‐I, GSEA results revealed the top 9 MDGs‐related pathways, including MAPK signaling pathway, the Wnt signaling pathway, cell cycle, as well as other cancer‐related pathways

The GSEA was exploited to investigate potential biological pathways that the 17‐methyalted drivers might be involved in, and we found a total of 79 items were enriched significantly with FDR < 0.25. The level of MDGs signature‐based risk score was defined as the phenotypes, and the result suggested that high‐risk level of MGDs may correlated highly with several important crosstalk, consisting of MAPK signaling pathway, Wnt signaling pathway, cell cycle, as well as other cancer‐related pathways (Figure 8).

## 4 DISCUSSION

Epigenetics, which focus on the features and modification of the genome, contains cytosine modifications of DNA, post‐translational modifications of histones, nucleosome positioning and interactions of spatial conformation between genomic regions and accessible genomic loci.^24^, ^25^ As a crucial part of epigenetic regulation, DNA methylation was reported to participate in malignant progression.^26^, ^27^ In BCa, Ahlén Bergman et al reported that high methylation level at the IFNG −4229 bp locus was associated with more advanced post‐cystectomy tumor stages, which might lead to a worse prognosis.^28^ Methylation also participated in the regulation of tumor suppressor mechanisms. For instance, methylation status of CpG islands was observed in the regulation of miR‐1‐3p expression, causing inhibition of the BCa cell proliferation, migration, and invasion.^29^ Although there are increasing studies concerning the methylation in BCa, limited research has explored the prognosis value on the genome‐wide screening of MDGs.

In this study, we analyzed the raw transcriptome data and screened out 228 MDGs. Then the ConsensusPathDB database was used for the analysis of biological functions of MDGs. The results revealed that most conspicuous pathways were transcriptional mis‐regulation pathways in cancer and EMT. After univariate Cox regression analysis and the establishment of the stepwise regression model, 17 epigenetic hub‐MDGs were identified. Furthermore, we calculated the risk score and assessed the predictive value of methylation signature in OS prediction, and found the satisfactory predictive efficiency of the risk signature by ROC. To further verificate the results above, we extracted high‐throughput sequencing data from the GEO database. The Kruskal‐Wallis test showed that higher risk score correlated with a higher level of TNM stage, high tumor grade, as well as advanced pathological stage. After screening of risk methylation loci with survival analysis and joint survival analysis, we examined enrichment of cancer‐related pathways associated with MDGs signature. The results above suggested that high‐risk level of MGDs may be correlated highly with several important crosstalk, consisting of the MAPK signaling pathway, the Wnt signaling pathway, cell cycle, as well as other cancer‐related pathways.

Keratins 8 (KRT8) is a key participation factor of keratinization, which exists abnormal performance in basal cell tumors. As representative of simple columnar epithelia, KRT8 is integral to epithelial differentiation.^30^ It is worth noting that abnormal epithelial‐mesenchymal transition is an important part of the malignant progression.^31^ Study has revealed that the high expression of KRT8 in gastric cancer is related to the upregulation of EMT pathway and might promote the tumor progression and metastasis.^32^ However, limited evidence has presented regulation mechanism of KRT8 and EMT signaling pathway in BCa. In our study, we identified KRT8 as a hub‐MDG, which might influence the BCa progression through EMT pathway analyzed by ConsensusPathDB database.

S100A16 belongs to S100 family, which is a superfamily of calcium‐binding proteins. S100A16, combining with S100A1‐S100A14 and S100A7, encodes in two tandem clusters on chromosome 1q21, which plays a powerful role in epidermal differentiation complex (EDC).^33^ As a novel detected S100 family member, S100A16 was observed over‐expressed in many malignancies.^34^ Nariaki et al demonstrated that S100A16 might increase the expression level of Oct4 and Nanog in cancer stem‐like cells in Yumoto human cervical carcinoma cells.^35^ According to our findings, S100A16 is highly expressed in bladder cancer, consistent with previous studies above.

TPM1 and TPM2 are both members of tropomyosin (TPM) family, which discovered as a category of actin binding proteins acting as inhibitors of cellular transformation.^36^ Previous research reported that overexpression of TPM1 was related to a larger tumor size and a higher tumor grade, and TPM1 was proved as a potential biomarker of renal cell carcinoma prognosis.^37^ Unlike TPM1, poorly TPM2 were identified associated with malignant progression prostate tumors.^38^ As filtrated MDGs, whether TPM1 and TPM2 interact in the malignant progression of bladder cancer remains further study.

To further explore the regulation of MDGs in bladder cancer, functional pathway analysis was performed and indicated that the 17 MDGs signatures were mainly associated with MAPK, Wnt and cell cycle signaling pathway, which is unique to our study. Studies indicated that methylation in malignant tumors might exert further regulatory functions through the above signaling pathways. In gastric cancer, SPG20 might be suppressed by methylation and result in activation of cell proliferation by upregulated the EGFR/MAPK pathway.^39^ Xiang et al demonstrated that zinc‐finger protein 545 is silenced by promoter methylation and acted as an inhibiting factor in colorectal cancer through the Wnt/β‐catenin, PI3K/AKT and MAPK/ERK signaling pathways.^40^ In colorectal cancer, it was also found that aberrant DNA methylation of WNT pathway genes might regulate tumor progression.^41^ In BCa, this study initially screened the signaling pathways associated with MDGs. Nevertheless, the specific mechanisms of action and regulation of these signaling pathways and methylation need to be further studied.

Finally, the risk model calculated based on 17 MDGs signatures has certain accuracy in assessing the prognosis of BCa patients, which is unique in our study. The AUC of ROC curve revealed the satisfactory predictive efficiency of the risk signature. What's more, Kruskal‐Wallis test showed that risk score had positive correlation with TNM stage, tumor grade and pathological stage (Stage III and IV), which functioned reasonably from clinical perspective and indicated its potential practical significance for BCa diagnosis and prognosis prediction.

Risk scores were calculated based on hub genes for evaluating the prognostic value, which was an advantage of our research. The AUC of the ROC based on risk score was 0.762 in our study. In previous studies, Zeng et al^42^ developed a novel nomogram with readily available clinicopathological information including grade, stage, age, lymph node, location, and histology for predicting cancer‐specific survival of upper tract urothelial carcinomas. However, the AUC of ROC was 0.74. In addition, Yin et al^43^ developed a nomogram based on 21‐miRNA signature. The AUC of ROC was only 0.663. The above results indicated that the risk score calculated based on the hub genes identified in our study might have a well‐prognostic value.

Despite the remarkable sense, it is inevitable that limitations also existed in our research. First, no clinical samples but only screening and verification were performed to extract target data through biological algorithm approaches. Secondly, data sources of BCa patients and validation are based on TCGA database and GEO database, which might result in selection bias due to ignorance of clinical and pathological data from BCa patients in other databases or clinical centers. Finally, these 17 hub‐MDGs should be further studied and validated to expound its specific regulatory function and mechanisms in BCa.

## 5 CONCLUSION

Our study indicated 17 hub‐MDGs by genome‐wide screening from public databases, calculated novel risk score and explored the prognostic value in BCa, which provided a promising approach to BCA prognosis assessment.

## CONFLICT OF INTEREST

The authors declare that the research was conducted in the absence of any commercial or financial relationships that could be construed as a potential conflict of interest.

## AUTHOR CONTRIBUTIONS

Jun Luo and Chuanjie Zhang designed the study and analyzed the data; Kangjie Shen and Chuanjie Zhang drafted the article; Yuxiao Zheng and Feng Qi was responsible for language correction. All authors finally approved the paper.
