# Supplementary figures and images for: Genome‐wide screening of abberant methylated drivers combined with relative risk loci in bladder cancer
Source: Cancer Med. 2019 Dec 3;9(2):768–82. doi: 10.1002/cam4.2665 (PMC6970050; doi:10.1002/cam4.2665)

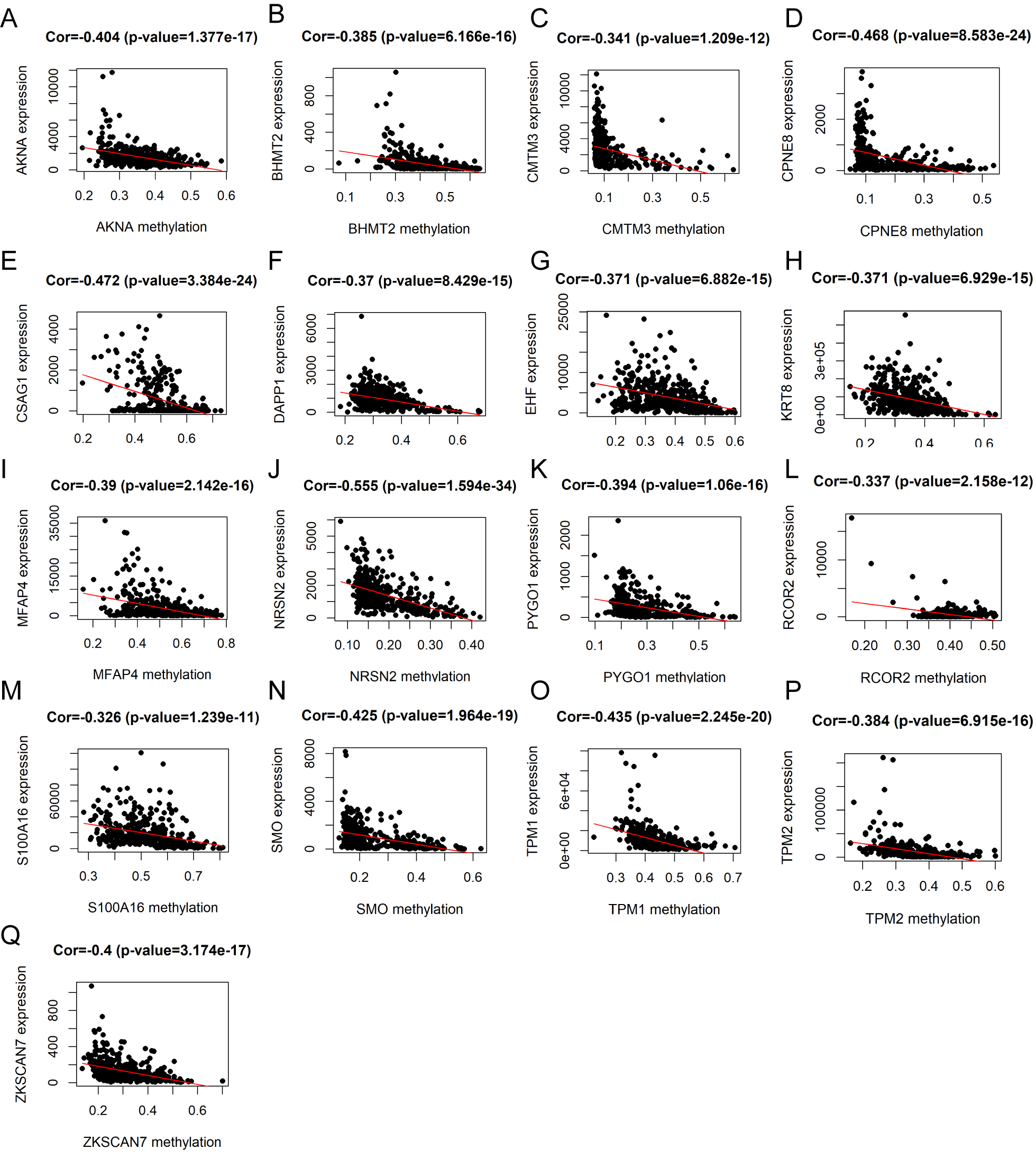

Supplement: Supplementary file 1 [file CAM4-9-768-s001.tif]

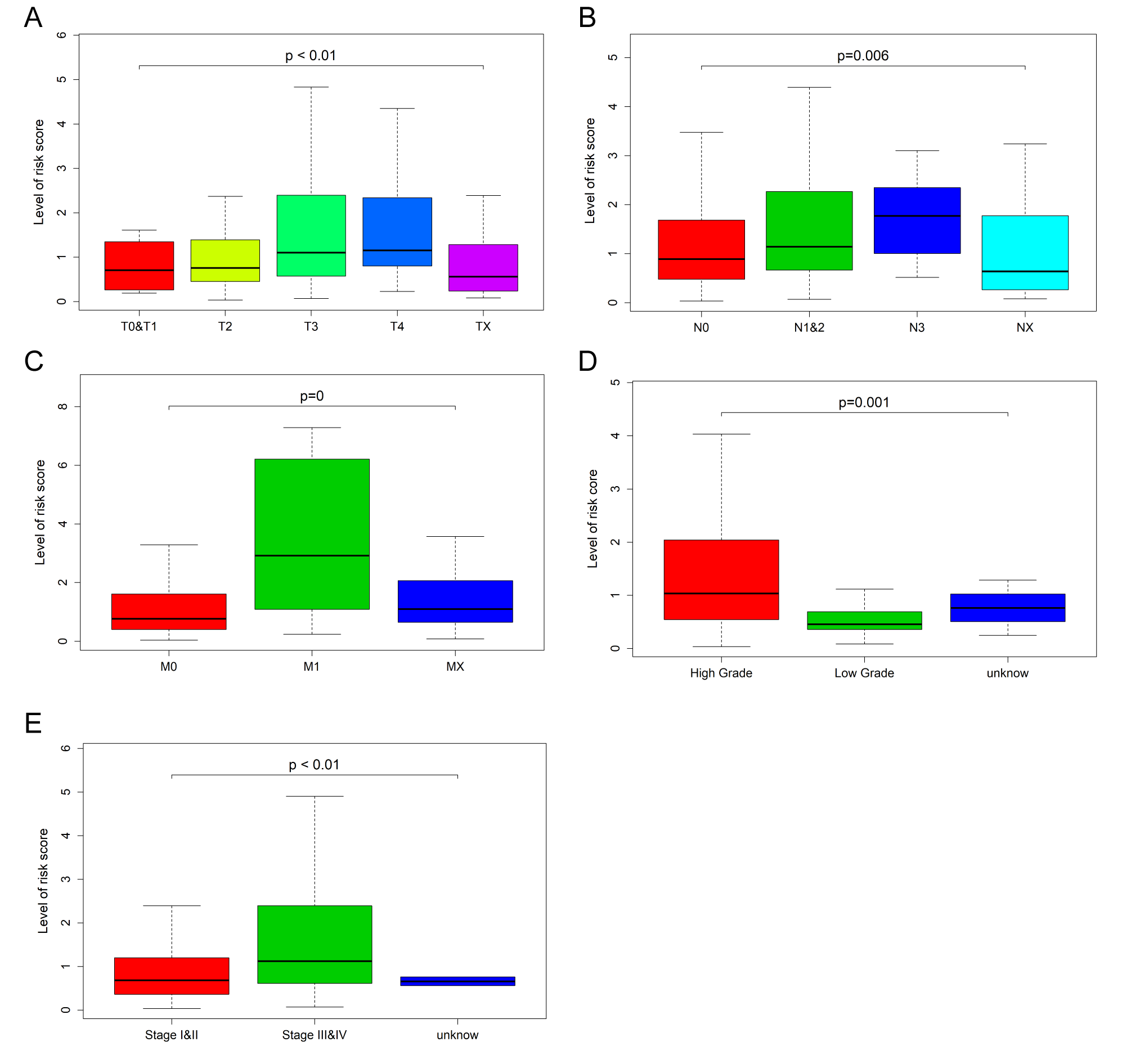

Supplement: Supplementary file 2 [file CAM4-9-768-s002.tif]

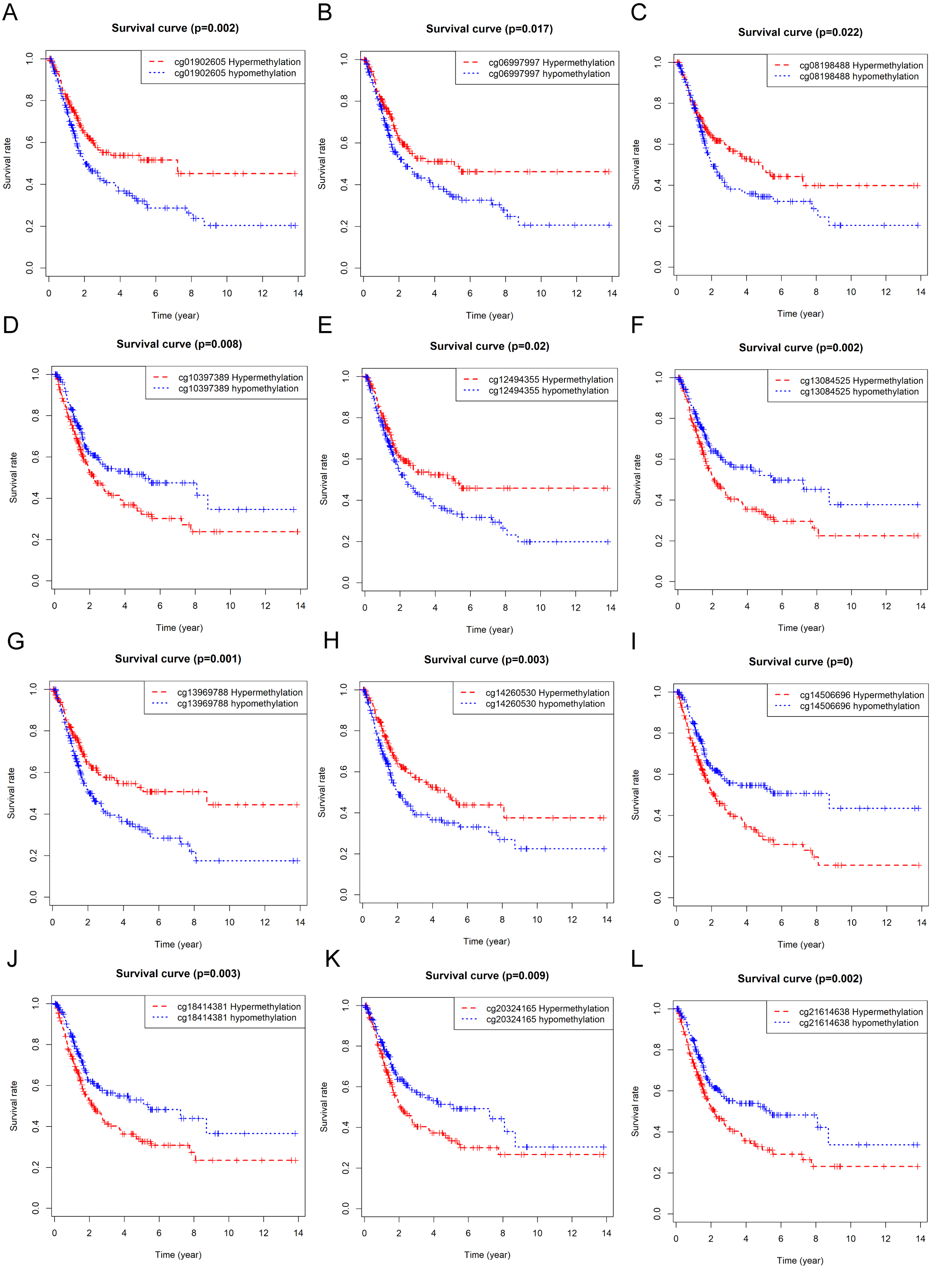

Supplement: Supplementary file 3 [file CAM4-9-768-s003.tif]
